# Long-Term Incidence of Stroke and Dementia in ASCOT

**DOI:** 10.1161/STROKEAHA.120.033489

**Published:** 2021-07-01

**Authors:** William N. Whiteley, Ajay K. Gupta, Thomas Godec, Somayeh Rostamian, Andrew Whitehouse, Judy Mackay, Peter S. Sever

**Affiliations:** 1 Centre for Clinical Brain Sciences, University of Edinburgh (W.N.W.).; 2 Nuffield Department of Population Health, University of Oxford (W.N.W.).; 3 William Harvey Research Institute, Queen Mary University of London (A.K.G.).; 4 National Heart and Lung Institute, Imperial College London (A.G., S.R., A.W., J.M., P.S.S.).; 5 London School of Hygiene and Tropical Medicine, University of London (T.G.).

**Keywords:** blood pressure, cholesterol, dementia, risk factors, stroke

## Abstract

Management of stroke risk factors might reduce later dementia. In ASCOT (Anglo-Scandinavian Outcome Trial), we determined whether dementia or stroke were associated with different blood pressure (BP)–lowering regimens; atorvastatin or placebo; and mean BP, BP variability, and mean cholesterol levels.

**Methods:**

Participants with hypertension and ≥3 cardiovascular disease risk factors were randomly allocated to amlodipine- or atenolol-based BP-lowering regimen targeting BP <140/90 mm Hg for 5.5 years. Participants with total cholesterol ≤6.5 mmol/L were also randomly allocated to atorvastatin 10 mg or placebo for 3.3 years. Mean and LDL (low-density lipoprotein) cholesterol, BP, and SD of BP were calculated from 6 months to end of trial. UK participants were linked to electronic health records to ascertain deaths and hospitalization in general and mental health hospitals. Dementia and stroke were ascertained by validated code lists and within-trial ascertainment.

**Results:**

Of 8580 UK participants, 7300 were followed up to 21 years from randomization. Atorvastatin for 3.3 years had no measurable effect on stroke (264 versus 272; adjusted hazard ratio [HR], 0.92 [95% CI, 0.78–1.09]; *P*=0.341) or dementia (238 versus 227; adjusted HR, 0.98 [95% CI, 0.82–1.18]; *P*=0.837) compared with placebo. Mean total cholesterol was not associated with later stroke or dementia. An amlodipine-based compared with an atenolol-based regimen for 5.5 years reduced stroke (443 versus 522; adjusted HR, 0.82 [95% CI, 0.72–0.93]; *P*=0.003) but not dementia (450 versus 465; adjusted HR, 0.94 [95% CI, 0.82–1.07]; *P*=0.334) over follow-up. BP variability (SD mean BP) was associated with a higher risk of dementia (per 5 mm Hg HR, 1.14 [95% CI, 1.06–1.24]; *P*<0.001) and stroke (HR, 1.21 [95% CI, 1.12–1.32]; *P*<0.001) adjusted for mean BP.

**Conclusions:**

An amlodipine-based BP regimen reduced the long-term incidence of stroke compared with an atenolol-based regimen but had no measurable effect on dementia. Atorvastatin had no effect on either stroke or dementia. Higher BP variability was associated with a higher incidence of later dementia and stroke.

## Abstract 2

Supplemental Digital Content is available in the text.

Although a reduction in stroke incidence earlier in life could prevent dementia in late life,^1^ clinical trials of stroke-preventing therapies do not show consistent reduction in the incidence of dementia.^2–4^ Therefore, longer follow-up to ages when dementia is more common might be needed to detect any effect.

The long-term follow-up of trials of blood pressure (BP) and LDL (low-density lipoprotein) cholesterol management provides an opportunity to test the hypothesis that intervention in earlier years reduces later dementia. Therefore, we sought to follow-up UK participants in ASCOT (Anglo-Scandinavian Cardiac Outcomes Trial). ASCOT was a 2×2 factorial randomized trial with 2 arms: a BP-lowering arm (BPLA) that compared amlodipine-based with atenolol-based BP-lowering regimens and a lipid-lowering arm (LLA) that compared atorvastatin with placebo.

In the original trial population, both interventions reduced stroke. Atorvastatin reduced all stroke (hazard ratio [HR], 0.73 [95% CI, 0.56–0.96]; *P*=0.024) after 3.3 years of follow-up, when this trial arm stopped early for evidence of efficacy.^5^ The amlodipine-based BP-lowering regimen compared with the atenolol-based regimen significantly reduced all stroke (HR, 0.77 [95% CI, 0.66–0.89]; *P*=0.0003) after a median follow-up of 5.5 years.^6^

In this article, the ASCOT follow-up is extended to 21 years and with new data ascertains both nonfatal and fatal stroke and dementia.^7^ We compare the incidence of stroke and dementia in UK participants allocated to either amlodipine-based or atenolol-based BP regimens (for median of 5.5 years) and atorvastatin or placebo (for median of 3.3 years). We estimate the association between in-trial (excluding the first 6 months) mean BP, BP variability, and mean total cholesterol with the subsequent incidence of stroke and dementia during follow-up.

## Methods

### Data Availability

Investigators wishing to access these data need to contract with NHS Digital and NHS Scotland, obtain the relevant ethical and data governance permissions, and have an analysis environment compliant with the Data Security and Protection Toolkit (www.dsptoolkit.nhs.uk). Other data from this study (code lists, statistical code) are available. Tabular data can be shared with collaborators if costs for further data extraction and analyses can be covered, by application to the ASCOT chief investigator P.S.

ASCOT was a 2×2 factorial trial based in both hospital clinics and primary care in the United Kingdom, Ireland, and Nordic countries that recruited participants between 1998 and 2002. The ASCOT BPLA^6^ included participants with hypertension (systolic BP ≥160 mm Hg if untreated or ≥140 mm Hg if treated or diastolic BP ≥100 mm Hg if untreated or ≥90 mm Hg if treated), who had no history of coronary heart disease, and ≥3 risk factors for cardiovascular disease (male sex, age ≥55 years, smoking, type 2 diabetes, peripheral artery disease, previous stroke or transient ischemic attack (TIA), left ventricular hypertrophy or other ECG abnormalities, microalbuminuria or proteinuria, ratio of plasma total cholesterol to HDL [high-density lipoprotein] cholesterol ≥6 mmol/L, or premature family history of coronary heart disease). Participants were randomly allocated to 2 unblinded BP-lowering regimens: amlodipine with or without perindopril (amlodipine based) or atenolol with or without bendroflumethiazide (atenolol based). At each follow-up visit, antihypertensive drug therapy was titrated to achieve a target BP of <140/90 mm Hg for nondiabetic patients and <130/80 mm Hg for diabetic patients, and information was recorded about adverse events and cardiovascular events.

The ASCOT LLA included participants from the BPLA trial who had a fasting total cholesterol of ≤6.5 mmol/L untreated with a cholesterol-lowering agent. Participants were randomly allocated to atorvastatin 10 mg or placebo.^5^

At the end of the period of active within-trial follow-up, we had no further information about BP or LDL cholesterol management.

We linked UK ASCOT participants from the date of trial entry to centrally held electronic health record (EHR) in Scotland, England, and Wales. We linked participants in England and Wales to death, hospitalization, and mental health records. In Scotland, we linked to death, hospitalization in general and mental health hospitals, and an audit of stroke care (from 2005). We only obtained data on those participants who had consented for long-term mortality follow-up and had not opted out of use of national datasets for research use.

### Measurement of BP and LDL Cholesterol

During active follow-up period of the trial (≈5.5 years), BP was measured in a sitting position after 5 minutes of rest 3× at screening, at a randomization appointment, at 1.5, 3, and 6 months, and subsequently 6 monthly thereafter. Total and LDL cholesterol were measured at 6 months and annually thereafter. LDL cholesterol was calculated using the LDL-Friedewald formula; in participants with triglyceride >4.5 mmol/L, LDL cholesterol was not estimated.

For each visit, we calculated a mean of the last 2 of 3 BP measures, or all measures if fewer were measured, from 6 months after randomization (by which time BP was stable) to last measured BP during the trial, in all participants with at least 3 visits for BP measurement and who were alive at the end of BPLA. We calculated a mean of these visit means and the SD of these means as a measure of visit-to-visit BP variability. Other measures of BP variability were closely correlated with SD in this dataset. We calculated mean total and LDL cholesterol from 6 months after randomization to end of BPLA. For observational analyses of cholesterol, we analyzed data from the participants in the BPLA who did not take part in the LLA (Figure I in the Data Supplement).

### Identification of Stroke and Dementia

Stroke was identified by investigators during the course of the trial and in EHR with previously validated code lists during and after the trial.^8^ Dementia was identified in EHR during and after the trial with previously validated code lists (Data Supplement).^9^ For all outcomes, we measured time to the first recorded outcome reported by either investigators or EHR. During the trial, stroke outcomes were adjudicated by a panel, but there was no further adjudication of stroke or dementia diagnoses recorded in EHR.

We use all stroke as the principal stroke outcome and where available analyzed ischemic stroke, hemorrhagic stroke (both subarachnoid hemorrhage and intracerebral hemorrhage), stroke of unspecified type, TIA, or retinal artery occlusion and all of these outcomes as a composite, cerebrovascular disease.

We used all dementia as the principal dementia outcome, and analyzed vascular dementia, Alzheimer disease, and unknown or rare dementia in mutually exclusive categories, as recorded in the EHR. No further adjudication of records was possible.

### Statistical Analyses

We analyzed all UK participants in ASCOT. Where linkage was not possible (largely because they had not consented to long-term mortality follow-up), we censored participants at the end of trial follow-up period.

We compared stroke and dementia incidence between participants in their allocated treatment groups and between participants with higher and lower baseline and mean LDL and total cholesterol and SD of BP.

We used Cox proportional hazards regression analysis to conduct survival analysis, tested the proportional hazards assumption, and reported the HR, its 95% CI, and *P*. Our principal analyses were for all-cause stroke and all-cause dementia. Censoring took take place on death, withdrawal of consent, date of end of linkage period, and out-migration. Follow-up time for observational analyses began at the end of trial, 5.5 years after randomization.

We report analyses unadjusted and adjusted for age, sex, baseline systolic BP, body mass index, age left education, history of diabetes, and smoking risk factors at baseline. We further adjusted SD of BP for mean BP analyses. We looked for evidence of effect modification by age, sex, ethnicity, baseline BP, total cholesterol, body mass index, and diabetes.

Stata 16 was used for all analyses.

### Ethical and Other Permissions

We obtained approval from the South East Scotland Research Ethics Committee (18/SS/0016), the Health Research Authority Confidentiality Advisory Group (18/CAG/0044), the Independent Group Advising on the Release of Data of NHS Digital, and the Public Benefit and Privacy Panel for Health and Social Care of NHS Scotland. We prepared this report with reference to the Reporting of Studies Conducted Using Observational Routinely Collected Health Data Statement (Table VII in the Data Supplement).^10^

## Results

**Figure 1.**
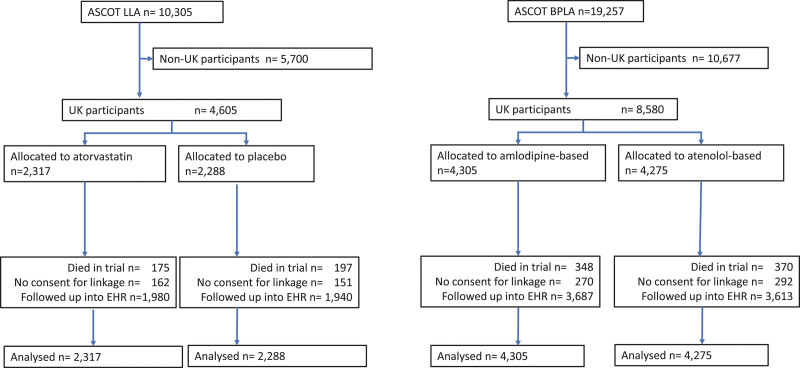
**Consolidated Standards of Reporting Trials diagram.** ASCOT BPLA indicates Anglo-Scandinavian Outcome Trial–Blood Pressure Lowering Arm; ASCOT LLA, Anglo-Scandinavian Outcome Trial–Lipid-Lowering Arm; and EHR, electronic health record.

In ASCOT, 8580 participants were from England, Wales, or Scotland, of whom 7300 were flagged at the end of the trial (Figure 1). Participants were followed up for a median of 17 years (interquartile range, 9–19) to a maximum of 21 years.

The UK participants were well matched by allocated group. On average, participants were 64 years of age (SD, 8) at trial entry, and the majority were men (81% in BPLA and 87% in LLA) and had left education before 16 years of age (79%). At baseline, BP was 162/92 mm Hg (SD, 18/10) and mean total cholesterol was 5.9 mmol/L (SD, 1.1) in BPLA and 5.5 mmol/L (SD, 0.8) in LLA. Participants had a history of diabetes (29%), stroke or TIA (12%), or other vascular disease (17%; Table 1). Compared with non-UK participants at baseline, UK participants were on average slightly older, less likely to smoke, drank more alcohol per week, with less time in education, and fewer had a history of vascular disease (Table I in the Data Supplement).

**Table 1.**
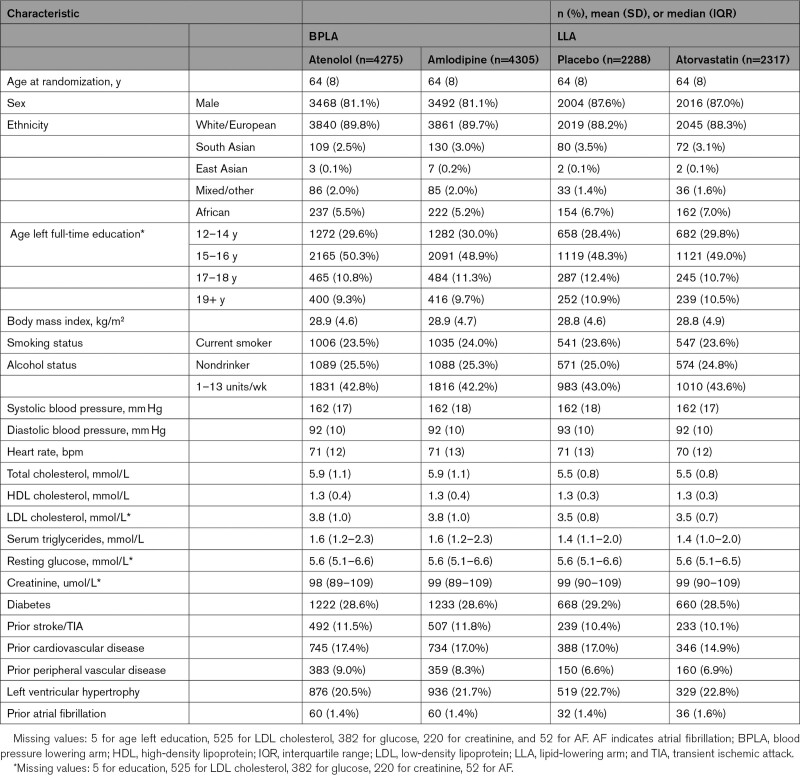
Baseline Characteristics of UK Participants in LLA and BPLA by Allocated Groups

From 6 months to the end of trial, participants allocated to an amlodipine-based regimen had a mean BP of 136 (SD, 10) mm Hg with a mean SD of all BP measurements of 11 mm Hg compared with, for the atenolol arm, 138 (SD, 11) mm Hg with a mean of SD of all measurements of 13 mm Hg.

During follow-up, 965 (11%) participants had a fatal or nonfatal stroke: 359 (4%) first strokes of uncertain type, 610 (7%) first ischemic strokes, 117 (1%) first intracerebral hemorrhages, 30 (<1%) first subarachnoid hemorrhages, 252 (3%) first TIAs, and 33 (<1%) first retinal artery embolisms (each participant could have >1 stroke type). Dementia was recorded in 915 participants: unknown type (381; 42%), vascular dementia (294; 32%), Alzheimer disease (221; 24%), and rare dementias (19; 2%). Nonfatal stroke during follow-up was associated with increased odds of later dementia (142/771 participants with stroke and 773/7809 participants with no stroke; OR, 2.05 [95% CI, 1.69–2.50] and *P*<0.001; adjusted OR, 1.67 [95% CI, 1.36–2.05] and *P*<0.001).

**Figure 2.**
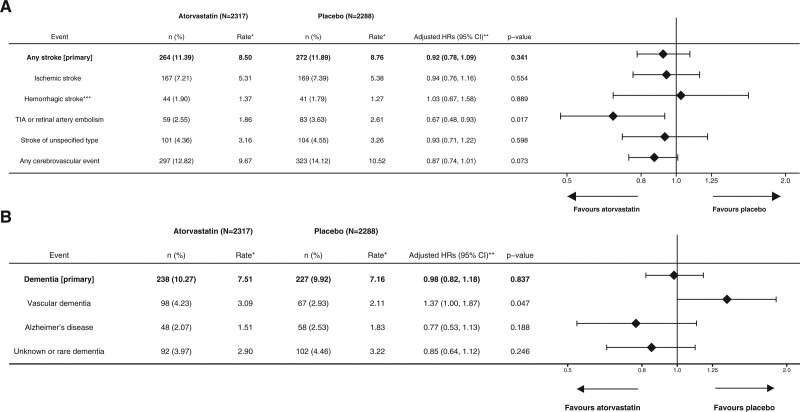
**Effect of allocation to atorvastatin or placebo in incidence of types of stroke and dementia. A**, Stroke; (**B**) dementia. HR indicates hazard ratio. *Per 1000 person-years. **Adjusted for baseline age, sex, systolic blood pressure, total cholesterol, body mass index, diabetes status, smoking habit, ethnicity, age left full-time education, and blood pressure lowering trial treatment allocation. ***Recorded intracerebral hemorrhage or subarachnoid hemorrhage. TIA indicates transient ischemic attack.

Participants allocated to atorvastatin rather than placebo for 3.3 years had nonsignificantly fewer fatal or nonfatal strokes during 21 years follow-up (272 placebo and 264 atorvastatin; adjusted HR, 0.92 [95% CI, 0.78–1.09]; *P*=0.341; Figure 2A; Figure II in the Data Supplement) There was no effect modification by any prespecified subgroup, different effect on different stroke types (interaction by stroke type *P*=0.907), or interaction with allocation to BP regimens (*P*=0.522; Table II in the Data Supplement).

Compared with participants allocated to placebo, those allocated to atorvastatin had a similar incidence of dementia (238 atorvastatin and 227 placebo; HR, 0.98 [0.82–1.18]; *P*=0.837; Figure 2B; Figure II in the Data Supplement), more due to vascular dementia (98 versus 67) than other dementia types (interaction by dementia diagnosis *P*=0.031). There was no modification by any prespecified subgroup or interaction with allocation to BP regimens (*P*=0.076) or after excluding cases in the first 10.5 years of follow-up (Table III in the Data Supplement).

There was no significant association after adjustment between baseline LDL cholesterol or total cholesterol or mean total cholesterol with stroke or dementia or any of the subtypes of either (Table 2).

**Table 2.**
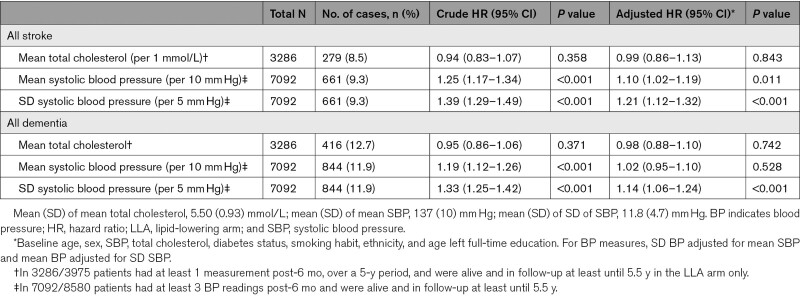
Observed Association Between Mean and SD of Blood Pressure and Mean Total Cholesterol Over the Course of the Trial and Later Development of Stroke or Dementia

**Figure 3.**
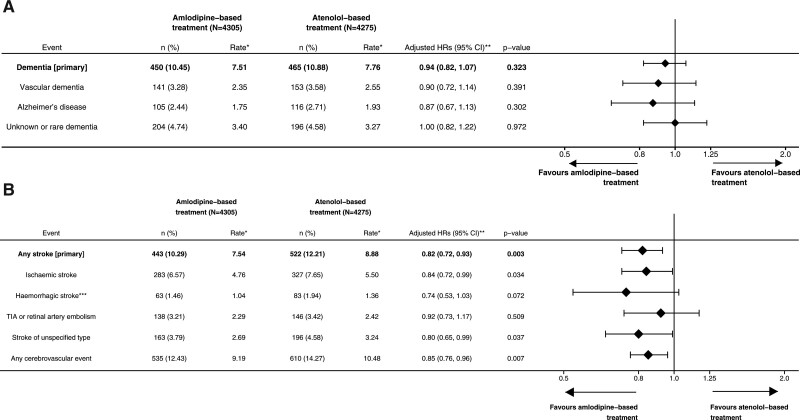
**Effect of allocation to amlodipine-based or atenolol-based regimen blood pressure (BP) lowering on incidence of (A) types and subtypes of stroke and (B) dementia.** HR indicates hazard ratio. *Per 1000 person-years. **Adjusted for baseline age, sex, systolic BP, total cholesterol, body mass index, diabetes status, smoking habit, ethnicity, age left full-time education, and blood pressure lowering trial treatment allocation. ***Recorded intracerebral hemorrhage or subarachnoid hemorrhage.

Participants allocated to an amlodipine-based rather than atenolol-based BP-lowering regimen for 5.5 years had a significant reduction in the incidence of fatal or nonfatal stroke during follow-up (443 amlodipine based and 522 atenolol based; HR, 0.82 [95% CI, 0.72–0.93]; *P*=0.003; Figure 3A; Figure III in the Data Supplement). The HR was the greatest for hemorrhagic strokes, although the CIs on each subtype overlapped (Figure 3A). There was no difference between within-trial versus post-trial period or within-trial stroke recorded by trial mechanisms alone or linked records alone. An amlodipine-based regimen reduced stroke significantly more where baseline total cholesterol was ≥6 mmol/L (*P*=0.019). There was no modification of the effect by other prespecified subgroups (Table IV in the Data Supplement) or by different stroke types (interaction by stroke type *P*=0.774).

There was no reduction in all dementia in participants allocated to an amlodipine-based BP regimen compared with an atenolol-based regimen (450 amlodipine and 465 atenolol; HR, 0.94 [95% CI, 0.82–1.07]; *P*=0.323; Figure 3B; Figure III in the Data Supplement). Effects did not differ by reported dementia type or prespecified subgroups (Table V in the Data Supplement) or after excluding cases in the first 10.5 years of follow-up.

After adjustment, a higher incidence of all stroke was observed with a 10-mm Hg higher mean systolic BP (adjusted HR, 1.19 [95% CI, 1.11–1.27]; *P*<0.011) and a 5-mm Hg higher SD in BP (HR, 1.21 [95% CI, 1.12–1.32]; *P*<0.001, adjusted in addition for mean BP; Table 2; Table VI in the Data Supplement).

After adjustment, there was no association between higher mean BP with later dementia (HR, 1.05 [95% CI, 0.99–1.14]), but there was evidence of reverse causality. After excluding dementia cases in the first 10.5 years of follow-up, mean BP was associated with dementia (HR, 1.13 [95% CI, 1.03–1.24]; *P*=0.009), though at 15.5 years, fewer cases were available (HR, 1.11 [95% CI, 0.96–1.27]; *P*=0.158). However, higher BP variability (SD of mean systolic BP) was associated with a higher incidence of later dementia (HR, 1.14 [95% CI, 1.06–1.24]; *P*<0.001) even after adjustment for mean BP, particularly for vascular dementia (HR, 1.34 [95% CI, 1.18–1.51]; *P*<0.001; Table 2; Table VI in the Data Supplement). This was not attenuated after adjustment for the occurrence of stroke between randomization and dementia diagnosis.

## Discussion

In this 20-year follow-up of ASCOT, an amlodipine-based BP-lowering regimen reduced the relative risk of stroke by 18% compared with an atenolol-based BP-lowering regimen. Although greater BP variability was associated with higher dementia incidence, and amlodipine-based regimens are associated with lower BP variability,^11^ we did not demonstrate that an amlodipine-based regimen for 5.5 years reduced dementia incidence after 20 years.

Participants allocated to 10-mg atorvastatin for a period of 3.3 years had no significant legacy of reduced stroke or dementia incidence over 20 years of follow-up. This study differs a study that did show a legacy effect on stroke because the trial period was shorter and the post-trial use of statins was greater.^12^

A legacy effect, that is a persistent protective effect of an intervention in a trial after it has stopped, is unusual for medications but has been observed in trials of statins and antihypertensives.^13,14^ Other than chance or bias, there are a number of potential causal mechanisms. There may be a cascade of benefit on stroke incidence after an early reduction in intermediate risk factors due to treatment, such as atrial fibrillation or endothelial damage. A reduction in disabling events during trial might reduce events after trial through effects on reductions in deprivation or other effects of disability on health.

In this study, mean BP at any time was not associated with dementia after accounting for the confounding effect of age. However, higher systolic BP is most strongly associated with dementia when measured many years before diagnosis,^15^ supported in our study by a significant association when mean BP was measured at least 10.5 years before a dementia diagnosis.

Greater BP variability was associated with higher dementia incidence particularly where the dementia type was recorded as due to vascular dementia. This is consistent with previous observations in cohorts recruited from community, clinical trials, and electronic records.^16–19^ Higher BP variability is largely due to age-related stiffening of large arteries and loss of baroreflex function. Antihypertensive drugs have little beneficial effect in reducing variability, although dihydropyridine calcium channel blockers may have a modest effect.^20^

A causal effect of higher BP variability on vascular dementia is plausible. We may not have detected an influence of amlodipine-based treatment on dementia incidence because of the short duration of treatment, the modest effect of amlodipine on BP variability within trial, or likely similar BP treatment between groups after the end of trial.

We did not observe an association between higher mean cholesterol and all stroke nor evidence of a differential association by stroke types. Observational associations between higher total cholesterol and risk of all stroke are largely neutral,^21^ although higher LDL cholesterol is associated with a higher incidence of all ischemic stroke and lower incidence of hemorrhagic stroke in more recent, large observational studies.^22^ The neutral association in this study may be because misclassification of stroke types in UK EHRs obscured the underlying relationship. The lack of an association between earlier total or LDL cholesterol with later dementia is consistent with previous observational analyses,^23^ although some analyses do demonstrate a positive association.^24^ The observed increase in vascular dementia with atorvastatin may be an effect of misclassification, or most likely chance, because the effect on dementia overall was neutral.

There are a number of limitations to our analyses.

First, we were unable to follow-up the entire ASCOT population because identifiers were not available for Scandinavian, Irish cohorts, or all UK participants. Therefore, there may have been imbalances in unmeasured confounders because randomization was not stratified by country. However, the sample size was large, and there was no evidence of imbalance in measured confounders. Adjustment of comparisons of allocated interventions for known confounders made almost no difference to the results.

Second, the duration of the interventions was short relative to the time to dementia, and post-trial management of BP and LDL cholesterol was likely to be similar between groups. If the randomly allocated treatments had continued for 20 years, then differences may have emerged, but such a study would not be practical. The interventions reduced all-cause mortality, stroke, and MI; however, higher doses of statin or more intense lowering of mean BP (as in the SPRINT-MIND trial [Systolic Blood Pressure Intervention Trial])^25^ or BP variability might lead to a reduction in dementia incidence.

Third, stroke and dementia were identified with EHR. There are 2 limitations with this approach: misclassification and underascertainment.

Misclassification was minimized with recommended code lists for dementia in any position and stroke in the first 2 positions of EHR or death records.^8,26^ Residual misclassification may still have existed (particularly for stroke in EHR), but we expect this to have been balanced between groups, and of note, the within-trial effect on stroke was similar using EHR only or adjudicated outcomes only. Dementia in EHR probably has substantial underascertainment, but the 20-year cumulative dementia incidence in this study (17%) was greater than population-based studies using in-person follow-up (eg, 20-year dementia incidence in men at 65 years of age is 7.7% in Framingham).^27^ In addition, the use of EHRs probably leads to less loss to follow-up than face-to-face or telephone follow-up. There may have been differences in classification of stroke or dementia over time, likely with increasing accuracy with more recent records, though there was no clear evidence of this in our analyses.

Fourth, although the association between stroke and dementia is strong (5-year cumulative poststroke dementia incidence of 33%^28^), most interventions modestly reduce symptomatic stroke incidence (and potentially minimal or asymptomatic strokes) and, therefore, expected reductions in dementia might be hard to detect in a study of thousands of people where only a small number experience a stroke.^29^ Therefore, despite the large number of dementia cases (915), this study may have been underpowered to detect a reduction in dementia incidence through this mechanism.

The long-term follow-up of a randomized trial is possible using national EHR. By including nonfatal events, the number of stroke was increased 5-fold from a previous analysis relying on deaths alone.^7^ However, current regulatory and data governance hurdles are substantial. Although the linkage was technically simple, overcoming these hurdles took several years.

By the end of the follow-up period, the predicted proportion of the population with dementia and with a history of stroke was similar, indicating that both disabling conditions are important to people with hypertension.

In conclusion, we demonstrate the importance of BP control with amlodipine rather than atenolol for stroke prevention and that starting amlodipine about 5 years earlier still has an important detectable effect on stroke incidence over 20 years. Despite this reduction in stroke in incidence, there was no reduction in dementia incidence, although dementia was almost as frequent as stroke over follow-up.

## Acknowledgments

We are grateful to the electronic Data Research and Innovation Service team (National Service Scotland), NHS Digital, and to participants in ASCOT (Anglo-Scandinavian Outcome Trial), particularly those who took part in the participants’ consultation group about this study and the ASCOT study co-ordinator.

## Sources of Funding

This study was funded by a Scottish Senior Clinical Fellowship from Chief Scienctist’s Office to Dr Whiteley (CAF/17/01) and Imperial College London. P. Sever is supported by the Biomedical Research Centre Award to Imperial College Healthcare NHS Trust and was a National Institute for Health Research Senior Investigator. The study funders played no role in study design; in the collection, analysis, and interpretation of data; in the writing of the report; or the decision to submit for publication. The corresponding author had full access to all the data in the study and had final responsibility for the decision to submit for publication.

## Disclosures

Dr Whiteley reports grants from the Chief Scientist’s Office during the conduct of the study, Alzheimer’s Society, British Heart Foundation, and UK Stroke Association outside the submitted work. P. Sever reports grants and personal fees from Amgen and Pfizer, Inc, during the conduct of the study; grants and personal fees from Amgen; and grants and personal fees from Pfizer, Inc, outside the submitted work. Dr Gupta reports other support from Servier outside the submitted work. The other authors report no conflicts.

## Supplemental Materials

Online Figures I–III

Online Tables I–VII

Online Spreadsheet

## Supplementary Material


