# Supplementary material for: Long-Term Incidence of Stroke and Dementia in ASCOT
Source: Stroke. 2021 Jul 1;52(10):3088–96. doi: 10.1161/STROKEAHA.120.033489 (PMC8478091; doi:10.1161/STROKEAHA.120.033489)
Supplement: Supplementary file 2 [file str-52-3088-s002.pdf]

SUPPLEMENTARY MATERIAL

Supplementary Figure I: cohort creation, observational analyses
Supplementary Figure II Cumulative incidence curves for UK participants allocated atorvastatin or placebo (a) time to first recorded stroke (b) first recorded dementia
Supplementary Figure III Cumulative incidence curves for UK participants allocated amlodipine-based or atenolol-based blood pressure management (a) time to first recorded stroke (b) first recorded dementia

Supplementary table I: Baseline characteristics for UK Legacy population and Scandinavian/Irish population
Supplementary table II Subgroup analyses HR of stroke comparing atorvastatin to placebo.
Supplementary table III Subgroup analyses HR of dementia comparing atorvastatin to placebo.
Supplementary table IV: Subgroup analysis: HRs for stroke comparing Amlodipine-based to Atenolol-based treatment
Supplementary table V: Subgroup analysis: HRs for the outcome of dementia comparing Amlodipine-based to Atenolol-based treatment
Supplementary table VI. Association between different blood pressure and LDL cholesterol measurements with stroke (a) and dementia (b)
Supplementary table VII: the RECORD checklist

Supplementary spreadsheet: phenotype definitions

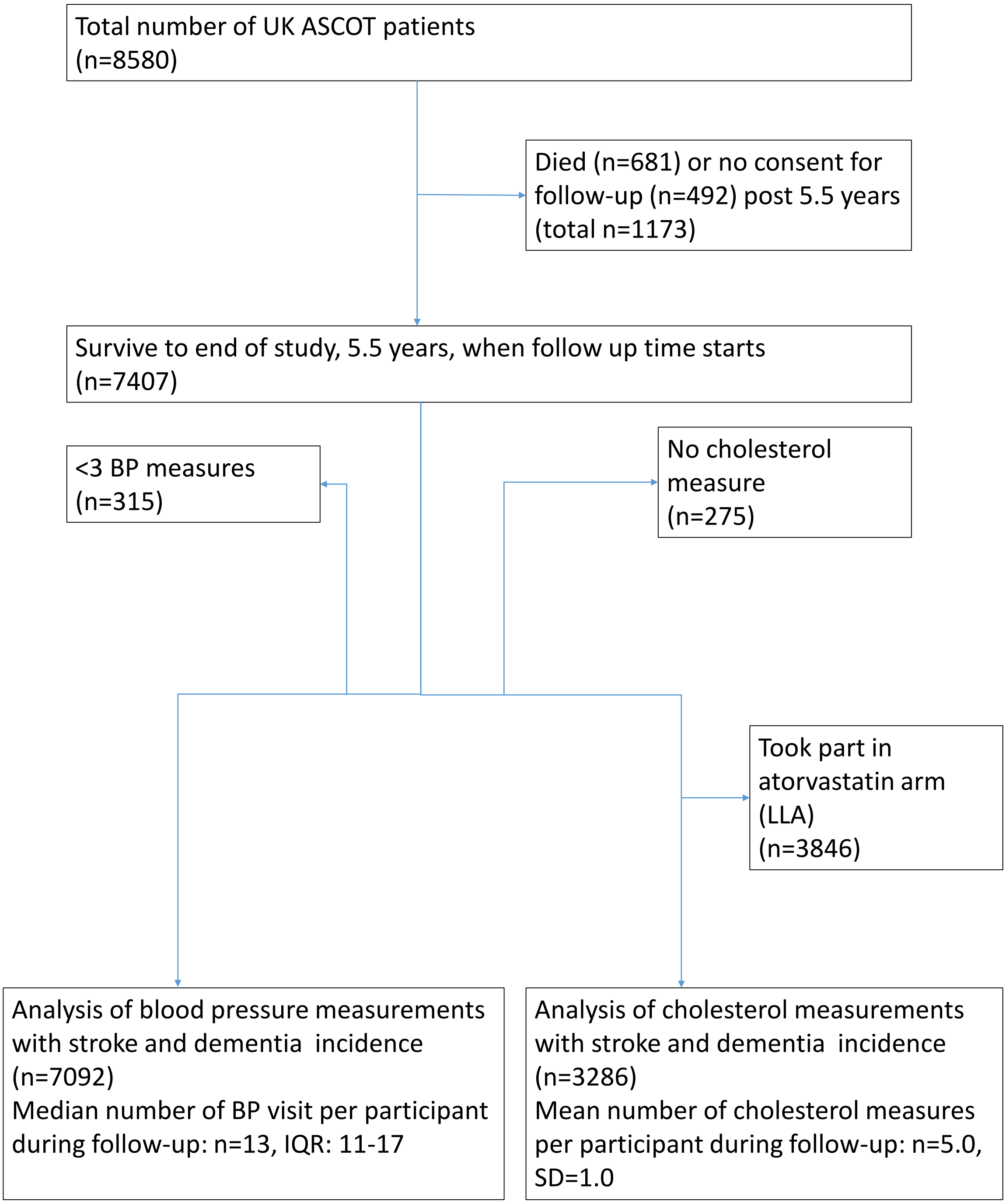

Figure I cohort creation, observational analyses

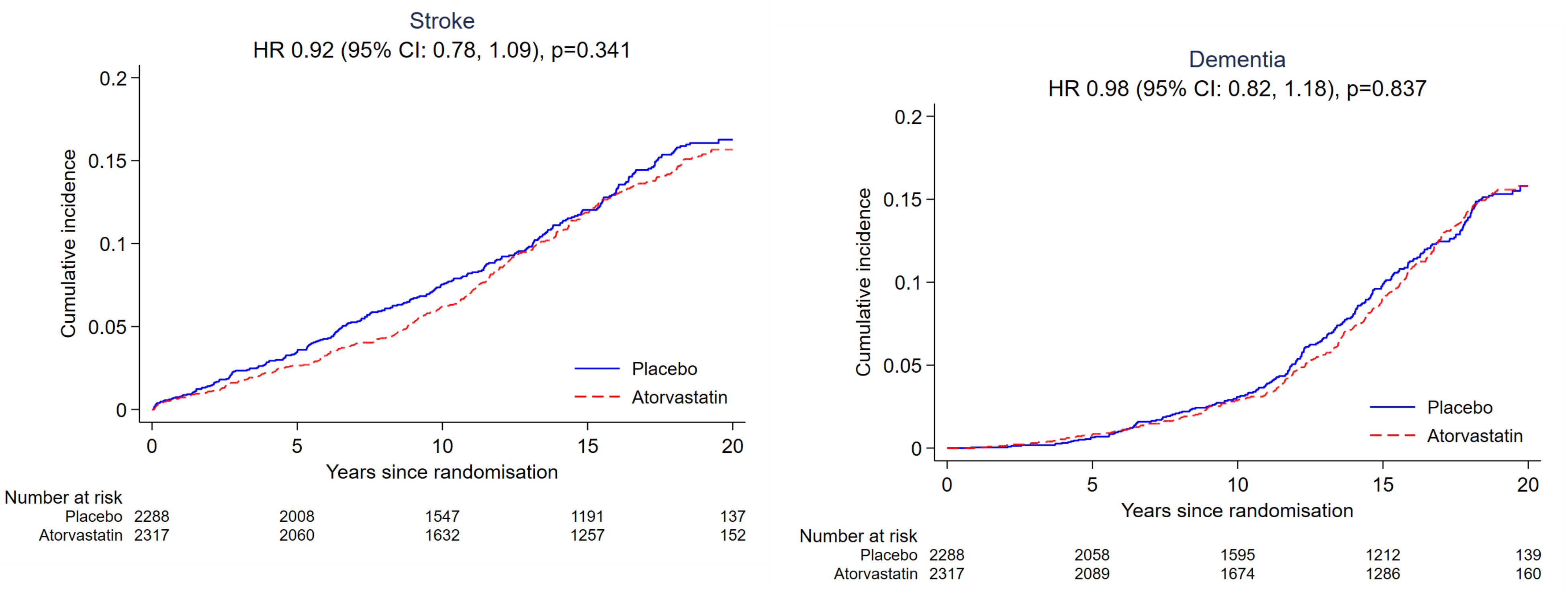

Supplementary Figure II Cumulative incidence curves for UK participants allocated atorvastatin or placebo (a) time to first recorded stroke (b) first recorded dementia

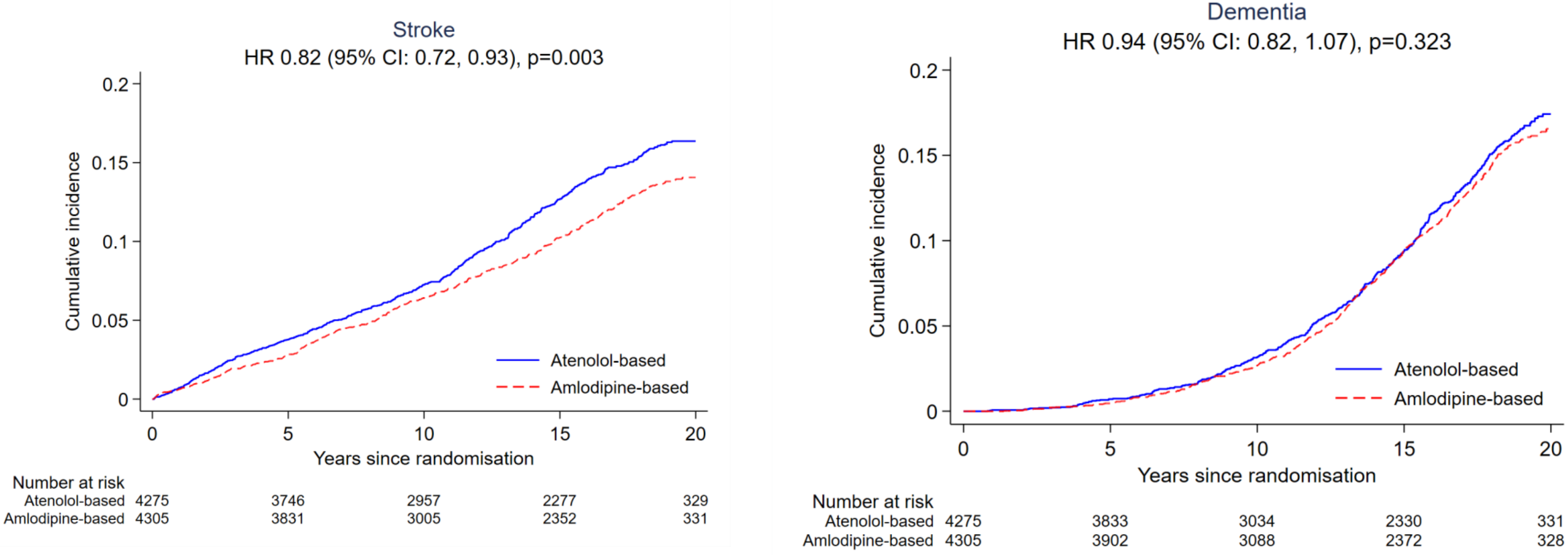

Supplementary Figure III Cumulative incidence curves for UK participants allocated amlodipine-based or atenolol-based blood pressure management (a) time to first recorded stroke (b) first recorded dementia

Supplementary table I: Baseline characteristics for UK Legacy population and Scandinavian/Irish population

Characteristic		n (%), mean (SD) or median (IQR)<br>UK participants (N=8580)	Non-UK participants (10677)
Age (years)		64 (8)	62 (9)
Gender	Male	6960 (81.1%)	7782 (72.9%)
Ethnicity	White/Europid	7701 (89.8%)	10656 (99.8%)
	South Asian	239 (2.8%)	7 (0.1%)
	Oriental	10 (0.1%)	6 (0.1%)
	Mixed/other	171 (2.0%)	4 (0.0%)
	African	459 (5.3%)	4 (0.0%)
Socioeconomic status*	12-14 years	2554 (29.8%)	3524 (33.0%)
	15-16 years	4256 (49.6%)	2749 (25.8%)
	17-18 years	949 (11.1%)	1555 (14.6%)
	19+ years	816 (9.5%)	2849 (26.7%)
	Missing	5	0
Body mass index (kg/m2)		28.9 (4.7)	28.6 (4.5)
Smoking status	Current smoker	2041 (23.8%)	3852 (36.1%)
Alcohol status	Non-drinker	2177 (25.4%)	2776 (26.0%)
	1-13 units per week	3647 (42.5%)	6591 (61.8%)
	14+ units per week	2756 (32.1%)	1300 (12.2%)
Systolic blood pressure (mmHg)		162 (18)	166 (18)
Diastolic blood pressure (mmHg)		92 (10)	97 (10)
Heart rate (bpm)		71 (12)	73 (13)
Total cholesterol (mmol/L)		5.9 (1.1)	5.9 (1.1)
HDL-cholesterol (mmol/L)		1.3 (0.4)	1.3 (0.4)
LDL-cholesterol (mmol/L)*		3.8 (1.0)	3.8 (1.0)
Serum triglycerides (mmol/L)		1.6 (1.2 to 2.3)	1.6 (1.2 to 2.3)
Resting glucose (mmol/L)*		5.6 (5.1 to 6.6)	5.6 (5.1 to 6.4)
Creatinine (umol/L)*		98 (89 to 109)	96 (87 to 106)
Diabetes mellitus		2455 (28.6%)	2682 (25.1%)
Prior stroke / TIA		999 (11.6%)	1114 (10.4%)
History of cardiovascular disease		1479 (17.2%)	3805 (35.6%)
History of peripheral vascular disease		742 (8.7%)	457 (4.3%)
History of left ventricular hypertrophy		1812 (21.1%)	2355 (22.1%)
Prior atrial fibrillation		120 (1.4%)	110 (1.0%)

Missing values : 1622 for LDL cholesterol, 1440 for glucose, 406 for creatinine, 90 for AF.

*age left full-time education

Supplementary Table II: Subgroup analysis: HRs for all stroke comparing atorvastatin to placebo

Subgroup		Number of subjects	Adjusted HR (95% CI)*	p-value	Interaction test p-value	Interaction p-value (with continuous variable modelled as continuous & linear)
Age	<60 years	1361	1.03 (0.68-1.55)	p=0.898		
	≥60 years	3244	0.91 (0.76-1.10)	p=0.317	p=0.598	p=0.637
Sex	Women	585	0.68 (0.45-1.01)	p=0.056		
	Men	4020	0.99 (0.82-1.20)	p=0.935	p=0.090	
Baseline SBP	<160 mmHg	2293	0.92 (0.71-1.19)	p=0.507		
	≥160 mmHg	2312	0.94 (0.75-1.17)	p=0.567	p=0.897	p=0.515
Baseline Total Cholesterol	<6 mmol/L	3304	0.93 (0.76-1.13)	p=0.453		
	≥6 mmol/L	1301	0.92 (0.66-1.29)	p=0.625	p=0.966	p=0.880
Baseline BMI	<20 kg/m²	55	1.54 (0.37-6.43)	p=0.558		
	20 - <25 kg/m²	842	0.72 (0.49-1.06)	p=0.100		
	25- <30 kg/m²	2093	1.12 (0.88-1.44)	p=0.359		
	≥30 kg/m²	1615	0.81 (0.60-1.08)	p=0.152	p=0.157	p=0.197
Baseline diabetes status	No diabetes	3277	0.94 (0.76-1.16)	p=0.582		
	Diabetes	1328	0.89 (0.67-1.19)	p=0.448	p=0.772	
Ethnicity	Black/ mixed	385	0.70 (0.38-1.32)	p=0.272		
	Asian	156	0.69 (0.23-2.11)	p=0.515		
	White	4064	0.95 (0.80-1.14)	p=0.603	p=0.574	

*adjusted for everything else listed in the table, smoking status at baseline, age left full-time education, & BPL treatment allocation

Supplementary table III: Subgroup analysis: HRs for the outcome of dementia comparing Atorvastatin to Placebo

Subgroup		Number of subjects	Adjusted HR (95% CI)*	p-value	Interaction test p-value	Interaction p-value (with continuous variable modelled as continuous & linear)
**Age**	**<60 years**	1361	0.98 (0.51-1.88)	p=0.947		
	**60-<65 years**	976	0.97 (0.60-1.57)	p=0.908		
	**65-<70**	1008	1.16 (0.83-1.63)	p=0.383		
	**≥70 years**	1260	0.91 (0.70-1.18)	p=0.473	p=0.733	p=0.332
**Sex**	**Women**	585	0.93 (0.58-1.49)	p=0.751		
	**Men**	4020	0.99 (0.82-1.21)	p=0.949	p=0.789	
**Baseline SBP**	**<160 mmHg**	2293	0.87 (0.66-1.13)	p=0.295		
	**≥160 mmHg**	2312	1.10 (0.86-1.40)	p=0.465	p=0.206	p=0.699
**Baseline Total Cholesterol**	**<6 mmol/L**	3304	1.08 (0.87-1.34)	p=0.480		
	**≥6 mmol/L**	1301	0.77 (0.54-1.09)	p=0.139	p=0.103	p=0.353
**Baseline BMI**	**<20 kg/m^2**	55	1.79 (0.45-7.16)	p=0.413		
	**20 - <25 kg/m^2**	842	0.93 (0.62-1.37)	p=0.698		
	**25- <30 kg/m^2**	2093	1.01 (0.78-1.31)	p=0.926		
	**≥30 kg/m^2**	1615	0.99 (0.69-1.42)	p=0.964	p=0.842	p=0.956
**Baseline diabetes status**	**No diabetes**	3277	0.98 (0.78-1.22)	p=0.824		
	**Diabetes**	1328	1.00 (0.72-1.38)	p=0.990	p=0.909	
**Ethnicity**	**Black/ mixed**	385	1.74 (0.99-3.05)	p=0.054		
	**Asian**	156	0.39 (0.08-1.92)	p=0.245		
	**White**	4064	0.93 (0.76-1.13)	p=0.464	p=0.062	

*adjusted for everything else listed in the table, smoking status at baseline, age left full-time education, & BPL treatment allocation

Supplementary table IV : Subgroup analysis: HRs for stroke comparing Amlodipine-based to Atenolol-based treatment

Subgroup		Number of subjects	Adjusted HR (95% CI)*	p-value	Interaction test p-value	Interaction p-value (with continuous variable modelled as continuous & linear)
Age	<60 years	2605	0.84 (0.62-1.15)	p=0.278		
	≥60 years	5975	0.82 (0.71-0.94)	p=0.005	p=0.869	p=0.967
Sex	Women	1620	0.83 (0.64-1.08)	p=0.159		
	Men	6960	0.82 (0.71-0.95)	p=0.008	p=0.937	
Baseline SBP	<160 mmHg	4362	0.87 (0.72-1.05)	p=0.139		
	≥160 mmHg	4218	0.80 (0.67-0.94)	p=0.009	p=0.525	p=0.333
Baseline Total Cholesterol	<6 mmol/L	4566	0.93 (0.79-1.10)	p=0.409		
	≥6 mmol/L	4014	0.70 (0.58-0.85)	p<0.001	p=0.031	p=0.019
Baseline BMI	<20 kg/m^2	83	1.68 (0.47-5.95)	p=0.424		
	20 - <25 kg/m^2	1512	0.70 (0.52-0.94)	p=0.016		
	25- <30 kg/m^2	3970	0.86 (0.71-1.03)	p=0.105		
	≥30 kg/m^2	3015	0.84 (0.67-1.05)	p=0.120	p=0.451	p=0.699
Baseline diabetes status	No diabetes	6125	0.75 (0.65-0.88)	p<0.001		
	Diabetes	2455	0.98 (0.79-1.22)	p=0.849	p=0.057	
Ethnicity	Black/ mixed	630	0.85 (0.55-1.31)	p=0.463		
	Asian	249	1.59 (0.60-4.18)	p=0.350		
	White	7701	0.81 (0.71-0.93)	p=0.002	p=0.399	

*adjusted for everything else listed in the table, smoking status at baseline, age left full-time education, & LL treatment allocation

Supplementary table V: Subgroup analysis: HRs for the outcome of dementia comparing Amlodipine-based to Atenolol-based treatment

Subgroup		Number of subjects	Adjusted HR (95% CI)*	p-value	Interaction test p-value	Interaction p-value (with continuous variable modelled as continuous & linear)
**Age**	**<60 years**	2605	0.75 (0.48-1.16)	p=0.196		
	**60-<65 years**	1856	0.93 (0.67-1.28)	p=0.641		
	**65-<70**	1881	0.92 (0.72-1.18)	p=0.519		
	**≥70 years**	2238	1.00 (0.82-1.20)	p=0.960	p=0.702	p=0.639
**Sex**	**Women**	1620	0.86 (0.66-1.11)	p=0.243		
	**Men**	6960	0.97 (0.83-1.12)	p=0.656	p=0.427	
**Baseline SBP**	**<160 mmHg**	4362	0.93 (0.77-1.12)	p=0.437		
	**≥160 mmHg**	4218	0.95 (0.79-1.13)	p=0.556	p=0.872	p=0.656
**Baseline Total Cholesterol**	**<6 mmol/L**	4566	0.88 (0.74-1.06)	p=0.178		
	**≥6 mmol/L**	4014	1.00 (0.83-1.20)	p=0.999	p=0.348	p=0.702
**Baseline BMI**	**<20 kg/m^2**	83	0.81 (0.26-2.47)	p=0.705		
	**20 - <25 kg/m^2**	1512	0.94 (0.71-1.25)	p=0.683		
	**25- <30 kg/m^2**	3970	0.99 (0.82-1.19)	p=0.930		
	**≥30 kg/m^2**	3015	0.86 (0.67-1.10)	p=0.236	p=0.832	p=0.515
**Baseline diabetes status**	**No diabetes**	6125	0.99 (0.85-1.16)	p=0.881		
	**Diabetes**	2455	0.84 (0.66-1.05)	p=0.131	p=0.241	
**Ethnicity**	**Black/ mixed**	630	1.33 (0.85-2.06)	p=0.209		
	**Asian**	249	0.89 (0.33-2.39)	p=0.814		
	**White**	7701	0.91 (0.79-1.04)	p=0.167	p=0.271	

*adjusted for everything else listed in the table, smoking status at baseline, age left full-time education, & LL treatment allocation

Table VI(a): Association of additional blood pressure and lipid measurements at baseline with all stroke

	**Crude HR (95% CI)**	**p-value**	**Adjusted HR (95% CI)***	**p-value**
Systolic blood pressure (per 10mmHg)	1.15 (1.11-1.19)	<0.001	1.07 (1.04-1.11)	<0.001
Diastolic blood pressure (per 10mmHg)	0.87 (0.81-0.93)	<0.001	1.04 (0.97-1.11)	0.244
Mean arterial pressure (per 10mmHg)	1.06 (1.00-1.13)	0.043	1.09 (1.03-1.16)	0.003
HDL-cholesterol (per mmol/L)	1.24 (1.05-1.47)	0.011	1.03 (0.85-1.24)	0.790
LDL-Cholesterol (per mmol/L)	0.91 (0.85-0.98)	0.009	0.90 (0.84-0.96)	0.002
Total Cholesterol (per mmol/L)	0.92 (0.87-0.97)	0.004	0.90 (0.85-0.96)	0.001
Triglycerides (per mmol/L)	0.86 (0.80-0.93)	<0.001	0.93 (0.86-1.01)	0.069

*Adjusted for age, sex, BMI, smoking status, diabetic status, ethnicity, SES

Table VI (b): Association of additional baseline blood pressure and lipid measurement at baseline with all dementia

	**Crude HR (95% CI)**	**p-value**	**Adjusted HR (95% CI)***	**p-value**
Systolic blood pressure (per 10mmHg)	1.13 (1.09-1.17)	<0.001	1.00 (0.96-1.04)	0.980
Diastolic blood pressure (per 10mmHg)	0.74 (0.69-0.80)	<0.001	0.97 (0.90-1.03)	0.321
Mean arterial pressure (per 10mmHg)	0.95 (0.89-1.01)	0.127	0.98 (0.92-1.04)	0.538
HDL-cholesterol (per mmol/L)	1.47 (1.25-1.72)	<0.001	1.11 (0.92-1.34)	0.283
LDL-Cholesterol (per mmol/L)	1.05 (0.98-1.12)	0.164	1.01 (0.94-1.09)	0.719
Total Cholesterol (per mmol/L)	1.03 (0.98-1.10)	0.262	1.01 (0.95-1.07)	0.830
Triglycerides (per mmol/L)	0.81 (0.75-0.88)	<0.001	0.92 (0.85-1.00)	0.065

*Adjusted for age, sex, BMI, smoking status, diabetic status, ethnicity, SES

Supplementary table VII The RECORD statement – checklist of items, extended from the STROBE statement, that should be reported in observational studies using routinely collected health data.

	Item No.	STROBE items	Location in manuscript where items are reported	RECORD items	Location in manuscript where items are reported
**Title and abstract**					
	1	(a) Indicate the study's design with a commonly used term in the title or the abstract (b) Provide in the abstract an informative and balanced summary of what was done and what was found	2	RECORD 1.1: The type of data used should be specified in the title or abstract. When possible, the name of the databases used should be included.<br><br>RECORD 1.2: If applicable, the geographic region and timeframe within which the study took place should be reported in the title or abstract.<br><br>RECORD 1.3: If linkage between databases was conducted for the study, this should be clearly stated in the title or abstract.	2
**Introduction**					
Background rationale	2	Explain the scientific background and rationale for the investigation being reported	3		3
Objectives	3	State specific objectives, including any prespecified hypotheses	3		3
**Methods**					
Study Design	4	Present key elements of study design early in the paper	3,4		3,4
Setting	5	Describe the setting, locations, and relevant dates, including	3,4		3,4

		periods of recruitment, exposure, follow-up, and data collection			
Participants	6	*(a) Cohort study* - Give the eligibility criteria, and the sources and methods of selection of participants. Describe methods of follow-up<br>*Case-control study* - Give the eligibility criteria, and the sources and methods of case ascertainment and control selection. Give the rationale for the choice of cases and controls<br>*Cross-sectional study* - Give the eligibility criteria, and the sources and methods of selection of participants<br><br>*(b) Cohort study* - For matched studies, give matching criteria and number of exposed and unexposed<br>*Case-control study* - For matched studies, give matching criteria and the number of controls per case	3,4	RECORD 6.1: The methods of study population selection (such as codes or algorithms used to identify subjects) should be listed in detail. If this is not possible, an explanation should be provided.<br><br>RECORD 6.2: Any validation studies of the codes or algorithms used to select the population should be referenced. If validation was conducted for this study and not published elsewhere, detailed methods and results should be provided.<br><br>RECORD 6.3: If the study involved linkage of databases, consider use of a flow diagram or other graphical display to demonstrate the data linkage process, including the number of individuals with linked data at each stage.	3,4
Variables	7	Clearly define all outcomes, exposures, predictors, potential confounders, and effect modifiers. Give diagnostic criteria, if applicable.	4,5,6	RECORD 7.1: A complete list of codes and algorithms used to classify exposures, outcomes, confounders, and effect modifiers should be provided. If these cannot be reported, an explanation should be provided.	4,5,6
Data sources/ measurement	8	For each variable of interest, give sources of data and details of methods of assessment (measurement).	4,5,6		4,5,6

		Describe comparability of assessment methods if there is more than one group			
Bias	9	Describe any efforts to address potential sources of bias	4,5,6		4,5,6
Study size	10	Explain how the study size was arrived at	4		4
Quantitative variables	11	Explain how quantitative variables were handled in the analyses. If applicable, describe which groupings were chosen, and why	5,6		5,6
Statistical methods	12	(a) Describe all statistical methods, including those used to control for confounding<br>(b) Describe any methods used to examine subgroups and interactions<br>(c) Explain how missing data were addressed<br>(d) *Cohort study* - If applicable, explain how loss to follow-up was addressed<br>*Case-control study* - If applicable, explain how matching of cases and controls was addressed<br>*Cross-sectional study* - If applicable, describe analytical methods taking account of sampling strategy<br>(e) Describe any sensitivity analyses	6		6
Data access and cleaning methods		..		RECORD 12.1: Authors should describe the extent to which the investigators had access to the database population used to create the study population.	7

				RECORD 12.2: Authors should provide information on the data cleaning methods used in the study.	
Linkage		..		RECORD 12.3: State whether the study included person-level, institutional-level, or other data linkage across two or more databases. The methods of linkage and methods of linkage quality evaluation should be provided.	7
**Results**					
Participants	13	(a) Report the numbers of individuals at each stage of the study (*e.g.*, numbers potentially eligible, examined for eligibility, confirmed eligible, included in the study, completing follow-up, and analysed)<br>(b) Give reasons for non-participation at each stage.<br>(c) Consider use of a flow diagram	Figure 1	RECORD 13.1: Describe in detail the selection of the persons included in the study (*i.e.,* study population selection) including filtering based on data quality, data availability and linkage. The selection of included persons can be described in the text and/or by means of the study flow diagram.	Figure 1
Descriptive data	14	(a) Give characteristics of study participants (*e.g.*, demographic, clinical, social) and information on exposures and potential confounders<br>(b) Indicate the number of participants with missing data for each variable of interest<br>(c) *Cohort study* - summarise follow-up time (*e.g.*, average and total amount)	Table 1		Table 1
Outcome data	15	*Cohort study* - Report numbers of outcome events or summary measures over time	Figure 3, Figure 5, Table 2		Figure 3, Figure 5, Table 2

		Case-control study - Report numbers in each exposure category, or summary measures of exposure<br>*Cross-sectional study* - Report numbers of outcome events or summary measures			
Main results	16	(a) Give unadjusted estimates and, if applicable, confounder-adjusted estimates and their precision (e.g., 95% confidence interval). Make clear which confounders were adjusted for and why they were included<br>(b) Report category boundaries when continuous variables were categorized<br>(c) If relevant, consider translating estimates of relative risk into absolute risk for a meaningful time period	Figure 3, Figure 5, Table 2		Figure 3, Figure 5, Table 2
Other analyses	17	Report other analyses done—e.g., analyses of subgroups and interactions, and sensitivity analyses	7,8,9		7,8,9
**Discussion**					
Key results	18	Summarise key results with reference to study objectives	9		9
Limitations	19	Discuss limitations of the study, taking into account sources of potential bias or imprecision. Discuss both direction and magnitude of any potential bias	11,12	RECORD 19.1: Discuss the implications of using data that were not created or collected to answer the specific research question(s). Include discussion of misclassification bias, unmeasured confounding, missing data, and changing eligibility over time, as they pertain to the study being reported.	11,12

Interpretation	20	Give a cautious overall interpretation of results considering objectives, limitations, multiplicity of analyses, results from similar studies, and other relevant evidence	12,13		12,13
Generalisability	21	Discuss the generalisability (external validity) of the study results	12,13		12,13
**Other Information**					
Funding	22	Give the source of funding and the role of the funders for the present study and, if applicable, for the original study on which the present article is based			
Accessibility of protocol, raw data, and programming code		16	16	RECORD 22.1: Authors should provide information on how to access any supplemental information such as the study protocol, raw data, or programming code.	16

*Reference: Benchimol EI, Smeeth L, Guttmann A, Harron K, Moher D, Petersen I, Sørensen HT, von Elm E, Langan SM, the RECORD Working Committee. The REporting of studies Conducted using Observational Routinely-collected health Data (RECORD) Statement. *PLoS Medicine* 2015; in press.

*Checklist is protected under Creative Commons Attribution (CC BY) license.
