# Supplementary figures and images for: Long-Term Incidence of Stroke and Dementia in ASCOT
Source: Stroke. 2021 Jul 1;52(10):3088–96. doi: 10.1161/STROKEAHA.120.033489 (PMC8478091; doi:10.1161/STROKEAHA.120.033489)

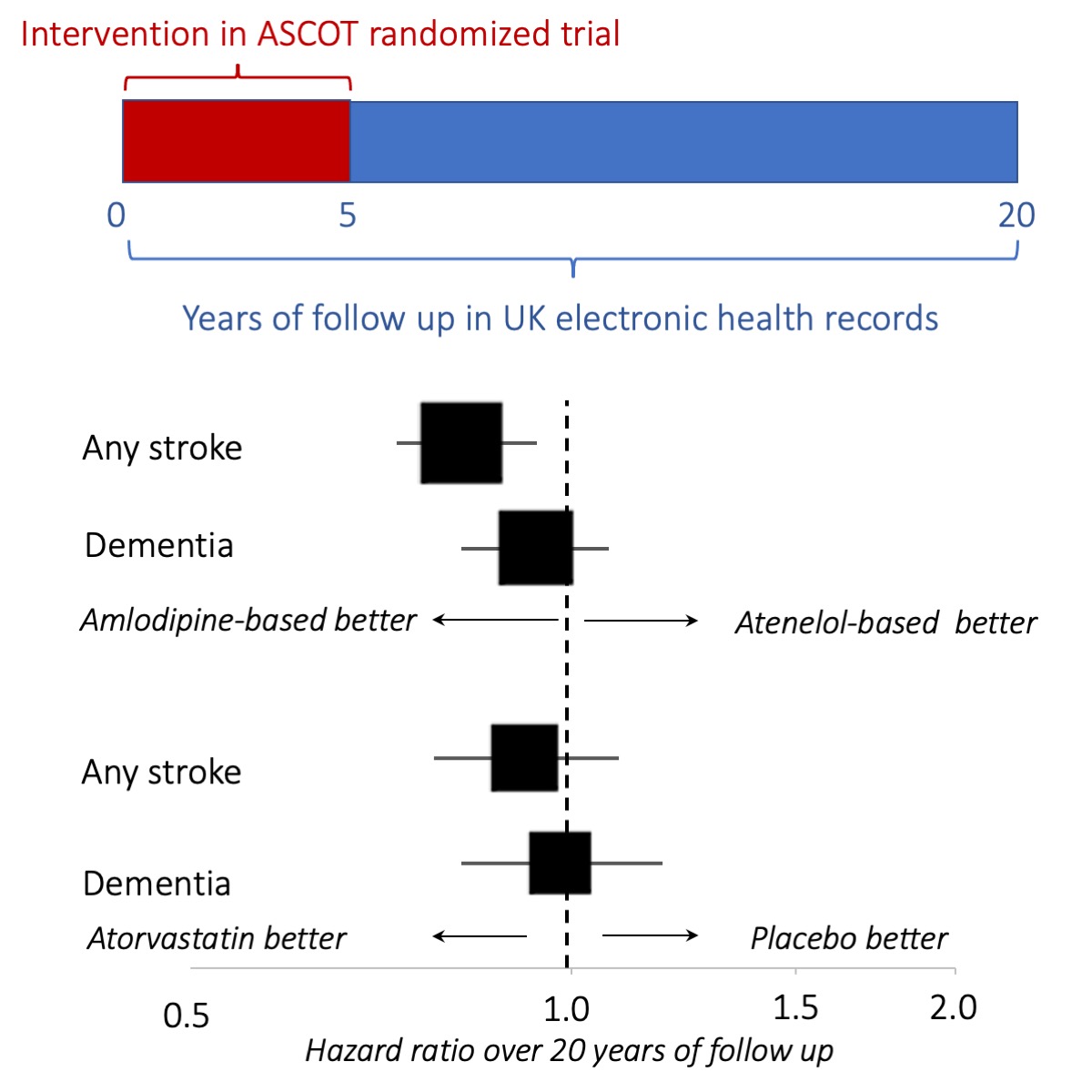

Supplement: Supplementary file 3 [file str-52-3088-s003.jpg]
